# Identification of blood immune and metabolic indicators explaining the variability of growth of pigs under contrasted sanitary conditions

**DOI:** 10.1186/s12917-021-02872-3

**Published:** 2021-04-15

**Authors:** N. Le Floc’h, F. Gondret, R. Resmond

**Affiliations:** PEGASE, INRAE, Institut Agro, 35590 Saint Gilles, France

**Keywords:** Growth rate, Inflammation, Metabolism, Pig (*Sus scrofa domesticus*), Prediction

## Abstract

**Background:**

Health and growth of pigs are affected by the hygiene of housing. Lower growth performance observed in poor hygiene of housing conditions is explained by reduced feed intake and metabolic changes caused by the activation of body defences. In a previous experiment, we reported contrasted average values of body weight gain, concentrations of circulating metabolites, redox and immune indicators in blood of pigs housed in good or poor hygiene conditions during the growing period. This study addressed inter-individual variability in these responses to determine whether a particular blood profile explains average daily gain (ADG) of the pig.

**Results:**

The data originated from 160 growing pigs, half of which subjected to a hygiene challenge for 6 weeks (W0 to W6) and the others housed in good hygiene conditions. Pigs originated from two lines divergently selected for residual feed intake (RFI). Individual body weights were recorded during this period, and relative ADG (rADG_W0-W6_) was calculated as the ADG corrected by the initial body weight measured at W0. Blood samples were taken before (W0) and 3 weeks (W3) after the beginning of the challenge. The analysed dataset consisted of 51 metabolites and indicators of immune and inflammatory responses measured on 136 pigs having no missing value for any variables, when calculated as the differences W3 minus W0 in circulating concentrations. An algorithm tested all possible linear regression models and then selected the best ones to explain rADG_W0-W6_. Six variables were identified across the best models and correlated with rADG_W0-W6_ with a goodness of fit (adjusted *R*^*2*^) of about 67%. They were changes in haptoglobin, global antioxidant capacity of plasma (Biological Antioxidant Power or BAP), free fatty acids, and 3 amino acids: leucine, tryptophan, and 1-methylhistidine. The effects of housing conditions and RFI lines were comprised in the variables of the selected models and none of these conditions improved accuracy of the predictive models, leading to genericity of the pinpointed metabolic changes in relation to variability of ADG.

**Conclusions:**

This approach allows us to identify blood variables, whose changes in blood concentrations correlated to ADG under contrasted sanitary conditions.

**Supplementary Information:**

The online version contains supplementary material available at 10.1186/s12917-021-02872-3.

## Background

In commercial farms, pig health status is influenced by animal internal factors (age, nutritional status, genetics …) and the presence of biotic (bacteria, fungi, virus …) and abiotic (gas, dust …) agents in the environment. Some of these agents are not intrinsically pathogenic but may become so when the organism fails to maintain its homeostasis. At the animal level, pig health is ensured through the continual cross-talk between the immune system and metabolism, both playing a pivotal role in animal coping abilities [1]. Indeed, the activation of the immune system and the inflammatory response observed in clinical and subclinical states are responsible for dramatic changes in metabolism leading to a repartitioning of nutrients and metabolites between growth and immune related functions. In a previous study, we reported contrasted average values for growth performance and concentrations in blood components related to metabolism and health status, between growing pigs selected for Residual Feed Intake (RFI, a measure of feed efficiency) and experimentally submitted to an inflammatory challenge based on poor hygiene of housing conditions for 6 weeks [2]. For some of these blood components, the changes in their circulating concentrations were maximal during the three first weeks of the challenge with 100 and 60% of increase in haptoglobin concentrations and blood neutrophil granulocytes, respectively. However, the maximum impact of the challenge on growth performance, measured as average daily gain (ADG), was observed after 6 weeks, with 25% decrease in ADG in pigs housed in poor hygiene compared to good hygiene. Altogether, the experimental design had generated a great variability in the concentrations of blood metabolites and immune indicators, and in pig growth performance, between and within housing conditions. We hypothesized that the range of variations in blood concentrations measured during the first 3 weeks may be correlated with the variation in ADG of the pigs during the 6 weeks of the challenge. Indeed, in a rat model of long-lasting catabolic state of sepsis induced by *Escherichia coli* IV injection, a significant correlation was reported between tumour necrosis factor-α concentration measured 1.5 h post-infection and body weight change recorded up to 10 days after infection [3]. This indicates that an early response to an immune challenge may be predictive of the long-term growth response. The aim of the present study was to select a subset of blood components whose variations in concentrations between two times, when combined in a linear regression, can be indicative of pig ADG variability, irrespective of the experimental factors, i.e. housing conditions and genetic lines. With this approach, we revisit the results of a factorial experimentation performed in controlled conditions with the objective to gain additional knowledge on the interrelations between health, growth and metabolism.

## Results

The database included 51 metabolites and immune indicators measured in blood sampled from growing pigs included in a 2 × 2 factorial design (Low RFI/High RFI (ATOL_0002160 [4]) and poor/good hygiene of housing conditions). Briefly, the main results of this experiment were that poor hygiene generated a systemic inflammation, increased the prevalence of pneumonia lesions and reduced ADG (ATOL_0000989) and feed efficiency (ATOL_0002159) compared to pigs housed in good hygiene [2]. The impact of poor hygiene on growth performance (ATOL_0002151) was greater at W6 with a difference in BW comprised between 4 and 10 kg on average for both lines, and 25% reduced ADG. The variations in their concentrations during the first 3 weeks of the trial (W0 and W3; [x]_W0-W3_ calculated as the concentration at W3 minus concentration at W0) were combined in linear regression models to explain the variability in the relative ADG of pigs calculated during the whole duration of the testing period (rADG_W0-W6_), irrespective of the experimental conditions. The best models were selected based on the value of Akaike Information Criteria (AIC). Fifteen models were initially retained to explain rADG, but four of them did not meet the multi-collinearity Variance Inflation Factor (VIF) threshold; therefore, they were discarded: two of them including leucine and isoleucine (ILE), one including valine and ILE and one model including red blood cell count and haematocrit. The remaining 11 linear models all had AIC values lower than AIC_best_model_ + 2 [5/36] and met multi-collinearity threshold (Table 1); adjusted *R*^*2*^ ranged from 0.677 to 0.672 and the models combined up to eight variables. Regression coefficients values for 13 variables that were included in at least one of the selected models are given in Table 1. None of the 11 selected models were over fitted, as indicated by stable metrics (Adjusted *R*^*2*^, Root Means Square Error, Mean Absolute Error) between each model and its 10-fold cross validation (Additional Table S1). This cross validation also showed that the regression coefficients were stable across 10-fold tests, and suggested that there were no outliers driving parameter estimate values. Residuals had similar values for the two fixed experimental factors (genetic line or hygiene of housing conditions) and their combinations (good housing conditions /Low RFI = 0.08 + − 3.83, good housing conditions /High RFI = 0.05 + − 4.19, poor housing conditions/Low RFI = 1.22 + − 4.24, poor housing conditions/High RFI = − 1.27 + − 3.45). Boxplots for residuals are available in the Additional Fig. S1. When added a posteriori to each model as explaining variables, these factors did not improve AIC or *R*^*2*^ of the models, suggesting that the effects of housing conditions and RFI lines were comprised in the variables for all of the selected models.

**Table 1:** The multiple regression coefficients, R-squared (*R*^*2*^) and adjusted R-squared of the 11 best models selected on the value of Akaike Information Criterion (AIC)

	Multiple regression coefficients for each variable (unit)^a^													Statistics		
Models	1-MH (μM)	BAP (μM)	dROM (CarrU)^b^	FFA (μM)	GLN (μM)	GRAN (10^3^/mm^3^)	HAPTO (g/L)	ILE (μM)	MONO (10^3^/mm^3^)	TRP (μM)	TYR (μM)	UREA (mg/L)	WBC (10^3^/mm^3^)	*R*^*2*^	adjusted *R*^*2*^	AIC
1	−1.224	0.013		0.007			− 1.232	−0.064	2.487	0.240		−0.011		0.701	0.677	617.92
2	−1.394	0.011	0.006	0.008	0.011		−1.594	−0.067		0.233				0.699	0.675	619.51
3	−1.198	0.014		0.007		0.147	−1.308	−0.065		0.241		−0.012		0.699	0.675	619.63
4	−1.336	0.011		0.007	0.008		−1.206	−0.068	2.180	0.230				0.699	0.675	619.73
5	−1.323	0.012		0.006			−1.376	−0.065	2.307	0.219				0.692	0.670	619.79
6	−1.384	0.011	0.004	0.006			−1.836	−0.064	2.469	0.226				0.697	0.673	618.86
7	−1.233	0.013		0.007			−1.165	−0.064		0.240		−0.010	0.070	0.697	0.673	618.88
8	−1.210	0.014		0.007			−1.115	−0.063		0.230		−0.010		0.691	0.670	618.88
9	−1.318	0.011		0.007	0.009		−1.083	−0.068		0.222				0.691	0.670	619.34
10	−1.238	0.013		0.008	0.007		−0.997	−0.066		0.236		−0.008		0.696	0.672	619.41
11	−1.277	0.012		0.008	0.009		−1.068	−0.066		0.264	−0.047			0.696	0.672	619.46

^a^The variables are changes in the concentrations in blood or plasma between two times W0 and W3 calculated as concentrations at W3 minus concentrations at W0 for plasma metabolites like AA (1-methylhistidine or 1-MH, glutamine or GLN, isoleucine or ILE, tryptophan or TRP and tyrosine or TYR), free fatty acids or FFA, and urea or UREA, plasma indicators of oxidative status (Biological Antioxidant Potential or BAP, Diacron reactive oxygen metabolites or dROM expressed in), acute phase protein (haptoglobin or HAPTO) and blood cells (neutrophil granulocytes (GRAN), monocytes (MONO) and white blood cells (WBC))

^b^CarrU = “Carratelli Units”, where 1 CARRU is equivalent to the oxidizing power of 0.08 mg H_2_O_2_/dL

All these 11 best models had very similar AIC value. In addition, six variables were common to all models. Seven other variables were present in only one up to five of these models; however, most of them were mathematically correlated. Therefore, we designed a minimal model combining only the six common variables (free fatty acid or FFA, Biological Antioxidant Potential or BAP, tryptophan or TRP, haptoglobin or HAPTO (ATOL_0000935), ILE, and 1-methylhistidine or 1-MH) to predict rADG_W0-W6_ (adjusted *R*^*2*^ = 0.664, AIC =620.36), instead of an averaged model for the 11 selected models. Descriptive statistics (Additional Table S2) and boxplots (Additional Fig. S2) for all the variables featured in at least one model, and for rADG_W0-W6,_ are available in supplementary files. The regression coefficients for the 11 individual models and the minimal model are presented in Tables 1 and 2, respectively. The standardized coefficients are also presented in Table 2. The minimal model was also checked for overfitting and stability with 10-fold cross-validation, and then cross-validated with the testing set. The metrics (adjusted *R*^*2*^, RMSE, MAE) are given in Table 3. The minimal model showed that with every increase of one standard deviation in FFA, rADG_W0-W6_ rose by 0.42 standard deviation, which were the strongest positive standardized coefficient in the model (standardized coefficients for BAP and TRP = 0.32 and 0.24 respectively). The minimal model also showed that with every increase of one standard deviation in 1-MH, rADG_W0-W6_ dropped by 0.35 standard deviation. For ILE and HAPTO, the standardized coefficients were - 0.33 and − 0.26, respectively.

**Table 2:** Multiple regression coefficients^a^ for the minimal model

Variables^b^	Regression coefficient	Std error	t value	Pr(>|t|)	Standardized coefficient
Intercept	28.9047	0.6664	43.3740	< 0.001	0.000
FFA	0.0061	0.0012	5.1530	< 0.001	0.424
BAP	0.0122	0.0023	5.3000	< 0.001	0.319
HAPTO	−1.2564	0.3317	−3.7880	< 0.001	−0.256
ILE	−0.0643	0.0156	−4.1340	< 0.001	−0.325
TRP	0.2106	0.0577	3.6530	< 0.001	0.245
1-MH	−1.3034	0.2136	−6.1020	< 0.001	−0.355

^a^Coefficient: multiple regression coefficient; Std error: Standard error of the coefficient; t-value: Student t test value; Pr(>|t|): *p*-value of the t test of nullity of the coefficient; Standardized Coefficient: Standardized coefficient of the multiple regression

^b^The variables are changes in the concentrations in plasma between two times W0 and W3 calculated as concentrations at W3 minus concentrations at W0 for 1-methylhistidine or 1-MH, tryptophan or TRP, isoleucine or ILE, free fatty acids or FFA, Biological Antioxidant Potential or BAP, haptoglobin or HAPTO

**Table 3:** Metrics for the minimal model (rADG_W0-W6_ ~ FFA + BAP + HAPTO + ILE + TRP + 1-MH)^a^ with the training set, its 10 folds repeated 10 times and with the testing set (validation set)

Models	*R*^*2*^	sd *R*^*2*^	*R*^*2*^ adjusted	sd *R*^*2*^ adjusted	RMSE	sd RMSE	MAE	sd MAE
Training set	0.683		0.664		3.87		3.187	
Training set k folds	0.648	0.176	0.629	0.131	4.1	0.813	3.422	0.698
Testing set	0.647		0.566		4.29		3.372	

No standard deviation can be estimated for the training and the testing set

*R*^*2*^ R-squared, *Sd R*^*2*^ Standard deviation of R-squared, *R*^*2*^ *adjusted* Adjusted R squared, *Sd* *R*^*2*^ *adjusted* Standard deviation of Adjusted R squared, *RMSE* Root Mean Square Error, *Sd RMSE* Standard deviation of Root Mean Square Error, *MAE* Mean Absolute Value, *Sd MAE* Standard deviation of Mean Absolute Error

^a^The model predicted relative ADG measured during the 6 weeks of the testing period (rADG_W0-W6_). The variables are changes in the concentrations in plasma between two times W0 and W3 calculated as concentrations at W3 minus concentrations at W0 for free fatty acids or FFA, Biological Antioxidant Potential or BAP, haptoglobin or HAPTO, isoleucine or ILE, tryptophan or TRP, 1-methylhistidine or 1-MH

## Discussion

A number of studies have described homeostatic regulation of plasma free amino acids (AA) and related metabolites [5] to help in maximizing protein accretion and growth of pigs, but few have tried to identify early plasma and/or blood markers that will predict animals susceptible to poor growth [6]. The present study enlightens a set of relevant blood immune and metabolic variables for whom range of variations between two times in an early period of growth (first 3 weeks of the test period) was able to explain the relative ADG during the whole test period (rADG_W0-W6_). During the whole test period, growing pigs were housed in two contrasted sanitary conditions that generated contrasted health and metabolic status. Considering differences in the measurements of concentrations in blood components between two times rather than at each time is motivated by the initial observation that the largest variations in immune and inflammatory traits were recorded between W0 and W3 of the trial. As an example, there were 100% increase in plasma haptoglobin and 60% increase in granulocyte count in blood between W0 and W3 between poor and good hygiene conditions [2], whereas these two indicators were measured at lower level thereafter. Our hypothesis was thus that the range of early changes could be correlated with the ADG measured during the whole duration of the challenge. At each time, blood was collected in the fasted state to ensure that, at least for metabolites, the level of feed intake and the time between the last meal and the blood collection did not influence the measured concentrations. In this study, it is impossible to decipher if variable was a cause or a consequence of rADG_W0-W6_ expressed by the pig, but the sign of the correlation is relevant to speculate on the mechanisms underlying modulation of growth performance in a context of poor sanitary conditions.

In this study, we used a multiple regression approach, so that each regression coefficient was the slope of the linear relationship between rADG_W0-W6_ (variable to be explained) and the part of a predictor variable that is independent of all other predictor variables. A positive regression coefficient (i.e., for BAP, FFA, and TRP) thus indicates that an increase (respectively, a decrease) in the concentration of a given metabolite was associated with increased (respectively, decreased) rADG_W0-W6_. Only the sign of the regression coefficient of variables in the model explaining rADG_W0-W6_ is considered because the variables are expressed in different units and even variables with the same unit have a different order of magnitude. Standardized coefficients can be also misleading as they distort the assessment of the effect of each variable by confounding the effect of the variable with the standard deviation of the predictor and the response variable [7].

A positive correlation between [BAP]_W0-W3_ and rADG_W0-W6_ was shown in the current study. The BAP measures the non-enzymatic antioxidant capability of plasma to reduce ferric ions to ferrous ions [8, 9]. It is usually interpreted in association to the measurement of dROM (Reactive Oxygen Metabolites derived compounds or Diacron reactive oxygen metabolites) that corresponds to the production of hydroperoxides [9]. In humans, both dROM and BAP are positively correlated [10] and their ratio may be used as an indicator of oxidative stress defined as the redox imbalance due to an excess of oxidants and a depletion of antioxidants [11]. In a previous study conducted in weaned piglets, piglets with high ADG had greater BAP and lower dROM concentrations than piglets with low ADG [12]. In our study, change in dROM was selected as a predictive variable for two out of 11 models, but it was not included in the minimal retained model contrary to change in BAP. Many studies conducted in growing pigs reported a positive correlation between endogenous antioxidant status and growth performance in pigs submitted to stressful conditions or fed with exogenous antioxidant compounds [13–15]. A positive correlation between [BAP] _W0-W3_ and rADG_W0-W6_ may be indicative of a better capacity to maintain antioxidant status as inflammatory and antioxidant status are usually negatively correlated [15, 16].

In support, the change in plasma concentrations of HAPTO had a negative contribution to rADG_W0-W6_. Haptoglobin is an inflammatory protein synthesized mainly by the liver in response to the stimulation by cytokines and more specifically by Interleukine-6 [17]. In the pig, HAPTO is considered as a minor or moderate acute phase protein, meaning that its plasma concentration increases moderately in response to a challenge [18]. Its concentration in plasma increases to reach a maximal concentration within 2 or 3 days [17] and lasts over 2 weeks [18]. Accordingly, on pig farms, plasma concentration of HAPTO increases in response to various situations, such as infectious and non-infectious diseases with clinical or subclinical symptoms, where health is deteriorated [19]. In these situations, higher HAPTO concentrations in plasma were associated with lower growth performance [2, 20].

In addition, [TRP]_W0-W3_ were positively correlated with rADG_W0-W6._ Besides being a component of body proteins as an AA, and a precursor of serotonin, TRP metabolism is closely related to the immune and inflammatory responses [21], since during inflammation, TRP is converted into kynurenine by the enzyme IDO located in immune cells like macrophages and dendritic cells. Tryptophan catabolism into kynurenine is thus known to be a mechanism regulating the immune response and oxidative stress. In pigs, inflammation reduced fasted plasma TRP concentrations as well as its availability for growth [22]. The positive regression coefficient observed for change in plasma TRP concentration may reflect a better capacity of pigs with higher growth rate to maintain their plasma tryptophan between W0 and W3, probably because of their better health status and reduced inflammation.

The variations in FFA, and two other AA, 1-MH and ILE, were also selected in the final model to explain rADG_W0-W6_. At the fasted state, FFA concentrations in plasma are usually interpreted as resulting from lipolysis and lipid mobilisation [23]. However, this interpretation might be simplistic since lipolysis mainly occurs in the adipose tissue where FFA can also be immediately recycled in triglycerides [24]. In our companion paper [2], irrespective of the experimental factors, a 50% drop of average fasted plasma concentrations in FFA between W0 and W3 was observed, meaning that [FFA]_W3-W0_ have negative values in most pigs considered in the present study. The physiological reason underlying this decrease remains elusive, but it may reveal a dramatic change in the capacity of pigs to release FFA during the first 3 weeks of the growing period. Finding [FFA]_W3-W0_ as a positive predictor of rADG_W0-W6_ suggests that pigs able to maintain a large capacity to mobilize their lipid reserves are then able to sustain more efficiently their growth needs. Indeed, lean growth rate is an energy consuming mechanism.

The literature on 1-MH is difficult to interpret since 1-MH (refered as Nτ-Methyl-L-histidine in the present paper) is often confused with 3-methylhistidine (Nπ-Methyl-L-histidine) [25]. The confusion comes from two ways of numbering the atoms in the imidazole ring of histidine, the nitrogen atom the closest to the side chain (π position) is generally designed as 1 by biochemists, and by 3 by organic chemists (and the reverse for the τ position). Both methylhistidines are constituents of the two dipeptides ophidine or balenine (ß-alanine-1-MH), and anserine (ß-alanine-3-methylhistidine), which are also designed as each other in the literature [25, 26]. Anserine and ophidine/balenine are produced in abundance in the muscle of different animal species from the methylation of carnosine (ß-alanyl-histidine), the most abundant peptide present in free form in the muscle [24]. The pig synthesizes both anserine and balenine [27]. In vitro, carnosine, anserine and balenine act as pH buffers and metal chelators and have antioxidant properties [27]. In pigs, 1-MH is largely retained in the muscle as balenine, making the measurement of 1-MH in urine not relevant for assessing muscle protein breakdown [28]. In the same species, 1-MH concentrations in blood and balenine content in the muscle increase with age, and thus, with muscle mass [28]. More studies are required to assess the biological meaning of plasma 1-MH concentration in relation to balenine in pig muscle, to confirm its relation with growth.

The latter AA included in the predictive model was ILE, which belongs to the group of branched chain AA (BCAA) with valine and leucine. These three AA share a common transamination pathway involving the enzyme branched chain AA transferase located in many tissues but the liver [29]. In pigs, the muscle is the major tissue involved in the transamination of BCAA [30]. Lower growth rate may be associated with lower transamination of ILE because of the lower mass of muscle. The reason why only ILE, and not valine and leucine, was selected as a potential indicator of growth was the fact that these variables are highly correlated between each other, leading to multicollineartity in models including these variables and their exclusion.

## Conclusion

The present study succeeded at selecting a limiting set of blood variables whose changes in blood between two experimental times are correlated to ADG of pigs. We used a multi regression approach to identify relationships between growth and blood indicators with a specific focus on indicators of metabolism and health. This approach confirms the well-known relationship between growth and HAPTO as a relevant indicator of health and inflammatory status, BAP, an indicator of antioxidant status, and TRP, an indispensable AA which metabolism is altered during inflammation, respectively. This study also reveals unexpected relationships between growth and changes in blood concentrations of other indicators, such as FFA, 1-MH and ILE. If the variables included in this study were obtained in controlled experimental conditions, we demonstrated that the two experimental factors did not modify our results, which is a first step towards genericity. The first output of the present study is to increase our knowledge on health and metabolism interconnections. Further studies and validation on larger dataset are of course required to open the way to the development of new early biomarkers of pig health and growth disturbances.

## Methods

### Ethics

The experiment was conducted at INRAE UE3P (Saint-Gilles, France) in accordance with the ethical standards of the European Community (Directive 2010/63/EU), and was approved by the Regional ethical committee (Comité Rennais d’Ethique en matière d’Expérimentation Animale, France: CREEA No. 07). The experiment received the authorization from the French Ministry of Higher Education, Research and Innovation. Pigs were born and reared at INRAE UE3P (Saint-Gilles, France). All pigs were slaughtered at the end of the experiment by electronarcosis and bleeding in the slaughter house of INRAE UE3P (Saint-Gilles, France).

### Animal and experimental design

The trial was described in details in Chatelet et al. [2] and included 160 Large-White pigs, 80 entire males and 80 females, produced from the 8th generation of two lines divergently selected for RFI. The RFI was calculated as the difference between the measured feed intake and feed intake predicted for maintenance and growth requirements [31]. The trial started after post-weaned pigs have been transferred to the experimental growing-finishing unit at 12 weeks of age, referred as Week 0 (W0; average weight 27.1 kg, SD: 3.5 kg). The experimental design consisted in a 2 × 2 factorial design (*n* = 40 pigs per group) including the two RFI lines (Low and High) housed in two contrasted hygiene conditions, good and poor, respectively. The poor housing conditions consisted of no cleaning and no sanitation of the room prior to occupancy by non-experimental pigs while good housing conditions were established using cleaned and disinfected rooms and strict biosecurity precautions. More details on how degradation of the environmental hygiene was induced and the impact on health and changes in animal performance could be found in [2]. The data used in this study were collected over 6 weeks (W0 to W6) for a total of 43 days +/− 1 day. Throughout the experimental period, pigs were fed ad libitum a growing diet formulated to meet nutritional requirements of growing pigs (as fed-basis: 9.47 MJ of net energy/kg, starch 44.2%; fat 3.1%; crude protein 15.3% and 8.3 g of digestible lysine (Lys), Lys/kg) and had free access to water.

### Origin of data

Pigs were weighed after an overnight fast at W0 (just before the transfer of pigs in experimental housing conditions), at W3 and at W6. Blood samples were collected the days of weighing before the morning meal. Blood cell count, plasma concentrations of HAPTO, glucose (ATOL_0000097), lactate, FFA, triglycerides, phospholipids, total cholesterol, β-hydroxybutyrate and urea (UREA), were measured on plasma prepared from blood collected on EDTA, whereas total protein, albumin, and AA concentrations were measured from plasma prepared from blood collected on heparin. The total number of leukocytes (White Blood Cell, WBC), red blood cells, hemoglobine, hematocrit, neutrophil granulocytes (GRAN), lymphocytes, as well as the count of platelets and monocytes (MONO) were determined on whole blood with an automatic cell counter MS 9.5 (Melet Schloesing Laboratories, Osny, France). Plasma HAPTO, glucose, lactate, FFA, triglycerides, phospholipids, total cholesterol, β-hydroxybutyrate, UREA, total protein, and albumin were measured by colorimetry on a Konelab 20i device (ThermoFisher Scientific, Courtaboeuf, France) using commercial kits. Free AA concentrations in plasma were measured with an ultra HPLC according to the Maastrak method (Waters UPLC Amino Acid Analysis AAA, LC system, Waters, Guyancourt, France) coupled to an UV detector [32]. A total of 30 AA were measured, among which 19 were proteinogenic AA. They included alanine, histidine, glycine, methionine, arginine, lysine, glutamine (GLN), asparagine, proline, serine, threonine, aspartate, glutamate, the three BCAA, leucine, ILE, valine, and the aromatic AA phenylalanine, tyrosine (TYR) and TRP. There were 11 non-proteinogenic AA, namely 1-MH, 3-methylhistidine, homocysteine, β-alanine, taurine, carnosine, α-amino-N-butyric and α-amino-adipic acids, hydroxyproline, ornithine and citrulline. Finally, hydroperoxides as dROM and the global antioxidant capacity of plasma or BAP were quantified [12].

### Data handling

Average daily gain (ADG) between W0 and W6 was calculated, and divided by the body weight (BW) measured at W0 to take into account the difference in initial BW to obtain rADG_W0-W6_ as the response variable. The maximum of the inflammatory response was reported to occur at W3 [2]. Thus, only blood formula and plasma concentrations measured at W0 and W3 were included in the database. Instead of the raw values of 51 metabolic and immune traits (Additional Table S2), their variation between W0 and W3, or [X]_W0-W3_, calculated as the difference W3 - W0, was used as explaining factors of rADG_W0-W6_. Pigs with at least one missing value for those 51 variables were removed from the database, so that 136 pigs (out of the 160) were finally included in the database. The database was randomly split into training and testing sets (respectively 80 and 20% of the database, 109 and 27 individuals, respectively). Several random seeds were tested to make the random split to ensure that initial experimental factors were well balanced within each set. The training set was used to select models and the testing sample was used as an external data set to validate these models.

### Data analysis

An exhaustive search of multiple linear regression models to explain rADG_W0-W6_ was first run on the training set using the R packages « glmulti » [33] and “leaps” [34]. This exhaustive search proceeded by a branch and bound algorithm (coded in the package “leaps”) which tested all possible models from a predictor subset, without actually running all candidate models. To achieve this, the algorithm constructs a search tree of all candidate models and then prunes it by using bounds on the objective function to minimise, thus reducing the number of possible models for the best model subsets [35]. Only first order models, i.e. models with no interaction, were tested, so that theoretically, 2^51^ models were built. The best models were then selected from the value of AIC [36] with the best model being the one with the lowest AIC. All models with an AIC value that felt in the range [AIC_best_model;_ AIC_best_model_ + 2] were kept [37]. All selected models were then checked for multicollinearity with the VIF [38] threshold fixed at five. Four models that did not meet this criterion were thus discarded. Then the models were checked with a k-fold cross validation procedure based on subsampling and resampling in which the training set is randomly split in 10 equal sized subsamples [39]. Nine of the subsamples (or folds) are used to fit the multiple regression, and the remaining fold is used as a testing set to evaluate the model. This cross-validation process is repeated 10 times so that all the folds are used once as a testing set. Metrics such as R squared, RMSE and MAE are averaged over the 10 replicates. The minimal model was validated with the testing set to ensure its predictive capability and stability, by comparing prediction metrics (R squared, RMSE, and MAE) obtained with the training and the testing set. The regression coefficients are stable across the folds indicating that they are not driven by outliers.

## Supplementary Information

**Additional file 1: Table S1.** Cross validation of 10 k-folds repeated 10 times for the 11 selected models to predict the relative Average Daily Gain (rADG_W0-W6_)^1^. **Table S2.** Values of the 51 blood variables and relative average daily gain^1^. **Fig. S1.** Boxplots for the residuals of the minimal model by hygiene of housing conditions and RFI lines (minimal model = rADG_W6-W0_ ~ FFA + BAP + HAPTO + ILE + TRP + 1-MH). The model predicted relative ADG measured during the 6 weeks of the testing period (rADG_W0-W6_). The variables are changes in the concentrations in plasma between two times W0 and W3 calculated as concentrations at W3 minus concentrations at W0 for free fatty acids or FFA, Biological Antioxidant Potential or BAP, haptoglobin or HAPTO, isoleucine or ILE, tryptophan or TRP, 1-methylhistidine or 1-MH. **Fig. S2.** Boxplots of variables present in at least one selected model. The variables are changes in the concentrations in plasma between two times W0 and W3 calculated as concentrations at W3 minus concentrations at W0 for free fatty acids or FFA, Biological Antioxidant Potential or BAP, haptoglobin or HAPTO, isoleucine or ILE, tryptophan or TRP, 1-methylhistidine or 1-MH, and relative average daily gain calculated between W0 and W6 or rADG_W0-W6._ Outliers (black points) for the boxplot are values outside the range Quartile(Q)3 + 1.5*interquartile-range (IQR), or Q1–1.5* IQR. Grey points represent the raw values for each variable, with a random noise added to better distinguish them on the X axis. Therefore, X axis has no particular meaning.

## Data Availability

Data are available on DataINRAE 10.15454/WDG7OD

## Abbreviations

[x]_W0-W3_: Difference in blood or plasma concentrations calculated as concentration at W3 minus concentration at W0
1-MH: 1-methylhistidine (Nτ-Methyl-L-histidine)
AA: Amino acid
ADG: Average daily gain
AIC: Akaike information criterion
BAP: Biological antioxidant power
BW: Body weight
dROM: Diacron reactive oxygen metabolites
EDTA: Ethylenediamine-tetra-acetic acid
FFA: Free fatty acids
GLN: Glutamine
GRAN: Neutrophil granulocytes
HAPTO: Haptoglobin
HRFI: High residual feed intake
ILE: Isoleucine
LPS: Bacterial liposaccharides
LRFI: Low residual feed intake
MONO: Monocytes
rADG_W0-W6_: Relative ADG
RFI: Residual feed intake
TRP: Tryptophan
TYR: Tyrosine
UREA: Urea
VIF: Variance inflation factor
W: Week
WBC: White blood cell count
