# Supplementary material for: Identification of blood immune and metabolic indicators explaining the variability of growth of pigs under contrasted sanitary conditions
Source: BMC Vet Res. 2021 Apr 15;17:166. doi: 10.1186/s12917-021-02872-3 (PMC8048059; doi:10.1186/s12917-021-02872-3)

**Additional files**

**Additional Table S1.** Cross validation of 10 k-folds repeated 10 times for the 11 selected models to predict the relative Average Daily Gain (rADG_W0-W6_)^1^

| **Models** | **R^2^** | **Sd R^2^** | **R^2^ adjusted** | **Sd R^2^.adjusted** | **RMSE** | **Sd RMSE** | **MAE** | **Sd MAE** |
| --- | --- | --- | --- | --- | --- | --- | --- | --- |
| mod 1 | 0.701 |  | 0.677 |  | 3.7577 |  | 3.0785 |  |
| mod 1 K-Folds | 0.656 | 0.176 | 0.638 | 0.131 | 4.0758 | 0.814 | 3.3793 | 0.714 |
| mod 2 | 0.699 |  | 0.675 |  | 3.774 |  | 3.0418 |  |
| mod 2 K-Folds | 0.662 | 0.168 | 0.644 | 0.122 | 4.0562 | 0.827 | 3.3415 | 0.746 |
| mod 3 | 0.699 |  | 0.675 |  | 3.7743 |  | 3.0763 |  |
| mod 3 K-Folds | 0.651 | 0.181 | 0.631 | 0.136 | 4.0853 | 0.85 | 3.385 | 0.745 |
| mod 4 | 0.699 |  | 0.675 |  | 3.7743 |  | 3.067 |  |
| mod 4 K-Folds | 0.657 | 0.179 | 0.638 | 0.134 | 4.0876 | 0.817 | 3.3746 | 0.72 |
| mod 5 | 0.692 |  | 0.67 |  | 3.8172 |  | 3.1285 |  |
| mod 5 K-Folds | 0.65 | 0.183 | 0.631 | 0.139 | 4.0939 | 0.804 | 3.3947 | 0.68 |
| mod 6 | 0.697 |  | 0.673 |  | 3.7835 |  | 3.0545 |  |
| mod 6 K-Folds | 0.654 | 0.18 | 0.635 | 0.135 | 4.0857 | 0.813 | 3.3499 | 0.696 |
| mod 7 | 0.697 |  | 0.673 |  | 3.7843 |  | 3.1175 |  |
| mod 7 K-Folds | 0.647 | 0.181 | 0.628 | 0.136 | 4.1067 | 0.85 | 3.4243 | 0.735 |
| mod 8 | 0.691 |  | 0.67 |  | 3.8201 |  | 3.1492 |  |
| mod 8 K-Folds | 0.652 | 0.172 | 0.633 | 0.127 | 4.0858 | 0.839 | 3.4133 | 0.731 |
| mod 9 | 0.691 |  | 0.67 |  | 3.8222 |  | 3.1204 |  |
| mod 9 K-Folds | 0.656 | 0.171 | 0.637 | 0.126 | 4.0776 | 0.818 | 3.382 | 0.743 |
| mod 10 | 0.696 |  | 0.672 |  | 3.7891 |  | 3.1048 |  |
| mod 10 K-Folds | 0.656 | 0.17 | 0.638 | 0.124 | 4.0788 | 0.843 | 3.4053 | 0.755 |
| mod 11 | 0.696 |  | 0.672 |  | 3.7901 |  | 3.1045 |  |
| mod 11 K-Folds | 0.657 | 0.169 | 0.638 | 0.123 | 4.0833 | 0.848 | 3.4033 | 0.755 |

^1^The variable to predict is relative ADG measured during the six weeks of the testing period (rADG_W0-W6_).

This table shows alternatively the metrics for the selected model and the metrics obtained for the same model but with 10 k-folds repeated 10 times

R^2^ : R-squared ; Sd R^2^ : Standard deviation of R-squared ; R^2^ adjusted : Adjusted R squared ; Sd R^2^.adjusted : Standard deviation of Adjusted R squared ; RMSE : Root Mean Square Error ; Sd RMSE : Standard deviation of Root Mean Square Error ; MAE : Mean Absolute Value ; Sd MAE : Standard deviation of Mean Absolute Error.

**Additional Table S2.** Values of the 51 blood variables and relative average daily gain^1^

| Variables | Abbreviation | Unit | Median | Mean | Standard-deviation | Min | Max |  |
| --- | --- | --- | --- | --- | --- | --- | --- | --- |
| Blood count |  |  |  |  |  |  |  |  |
| Red blood cells |  | 10^6^/mm^3^ | 0.40 | 0.35 | 0.58 | -2.17 | 2.1 |  |
| Hematocrit |  | % | 1.65 | 1.30 | 3.44 | -13.0 | 9.80 |  |
| Hemoglobin |  | g/dL | 0.62 | 0.53 | 0.91 | -3.70 | 2.75 |  |
| White blood cells | WBC | 10^3^/mm^3^ | 3.70 | 4.39 | 7.89 | -13.4 | 36.7 |  |
| Lymphocytes |  | 10^3^/mm^3^ | 1.20 | 1.58 | 4.74 | -14.3 | 32.6 |  |
| Monocytes | MONO | 10^3^/mm^3^ | 0.10 | 0.15 | 0.29 | -0.95 | 1.20 |  |
| Neutrophile granulocytes | GRAN | 10^3^/mm^3^ | 1.85 | 2.65 | 4.94 | -9.85 | 26.5 |  |
| Platelets |  | 10^3^/mm^3^ | -150.0 | -184.5 | 150.5 | -618.0 | 167.0 |  |
| Inflammation |  |  |  |  |  |  |  |  |
| Haptoglobin | HAPTO | g/L | 0.98 | 1.00 | 1.35 | -2.84 | 5.66 |  |
| Oxydative balance |  |  |  |  |  |  |  |  |
| dROM | dROM | CarrU^2^ | 115.5 | 106.4 | 187.9 | -444.6 | 724.7 |  |
| BAP | BAP | µM | 53.6 | 56.0 | 184.3 | -431.9 | 658.3 |  |
| Plasma metabolites |  |  |  |  |  |  |  |  |
| Glucose |  | mg/L | 109.0 | 97.5 | 179.2 | -352.5 | 883.2 |  |
| Lactate |  | µM | 361.5 | 726.5 | 4769.6 | -11664.2 | 14380 |  |
| Free fatty acids | FFA | µM | -516.4 | -518.7 | 472.0 | -1673.7 | 833.2 |  |
| Cholesterol |  | mg/L | -79.0 | -105.3 | 187.9 | -646.3 | 239.6 |  |
| Hydroxybutyrate |  | µM | -0.50 | 5.01 | 43.7 | -94.1 | 297.1 |  |
| Phospholipids |  | g/L | -0.12 | -0.12 | 0.23 | -0.74 | 0.47 |  |
| Triglycerides |  | mg/L | -235.0 | -290.4 | 328.5 | -1680.1 | 432.5 |  |
| Albumin |  | g/l | -1.34 | -1.50 | 3.14 | -9.51 | 8.79 |  |
| Proteins |  | g/L | 3.48 | 4.69 | 6.8 | -11.6 | 50.9 |  |
| Urea | UREA | mg/L | -40.7 | -35.9 | 84.7 | -236.2 | 167.0 |  |
| Ammonia |  | µM | 48.1 | 12.8 | 147.1 | -540.3 | 394.5 |  |
| α-aminobutyric acid |  | µM | -4.49 | -6.80 | 14.5 | -40.4 | 26.2 |  |
| α-amino adipic acid |  | µM | -22.13 | -24.1 | 36.6 | -110.0 | 82.9 |  |
| Alanine |  | µM | 25.4 | 17.8 | 125.6 | -330.0 | 374.8 |  |
| Arginine |  | µM | -11.9 | -9.33 | 28.9 | -77.6 | 67.9 |  |
| Asparagine |  | µM | 1.26 | 0.87 | 6.97 | -34.2 | 17.8 |  |
| Aspartate |  | µM | 2.46 | 2.72 | 7.97 | -22.2 | 20.1 |  |
| β-alanine |  | µM | -6.66 | -6.54 | 4.83 | -18.7 | 13.6 |  |
| Carnosine |  | µM | 2.02 | 2.52 | 7.11 | -15.5 | 34.1 |  |
| Citrulline |  | µM | -10.9 | -11.8 | 13.6 | -58.9 | 15.3 |  |
| Glutamine | GLN | µM | 34.6 | 42.3 | 85.6 | -268.2 | 277.5 |  |
| Glutamate |  | µM | 32.2 | 38.6 | 88.6 | -239.4 | 201.9 |  |
| Glycine |  | µM | 128.9 | 141.2 | 195.4 | -438.4 | 864.3 |  |
| Histidine |  | µM | 4.59 | 5.45 | 13.95 | -25.22 | 43.53 |  |
| Hydroxy-Proline |  | µM | -11.3 | -11.8 | 21.4 | -61.2 | 39.1 |  |
| Isoleucine | ILE | µM | -16.9 | -17.7 | 34.9 | -104.2 | 88.7 |  |
| Leucine |  | µM | -11.0 | -16.4 | 58.6 | -157.3 | 161.8 |  |
| Lysine |  | µM | 3.94 | 3.27 | 52.9 | -133.4 | 137.1 |  |
| Methionine |  | µM | 2.76 | 1.81 | 8.12 | -21.1 | 24.1 |  |
| Ornithine |  | µM | -4.54 | -4.75 | 13.6 | -51.8 | 25.4 |  |
| Phenylalanine |  | µM | -7.97 | -7.81 | 20.7 | -56.1 | 46.9 |  |
| Proline |  | µM | 13.0 | 12.4 | 31.3 | -84.5 | 116.4 |  |
| Serine |  | µM | -4.95 | -6.55 | 29.2 | -92.2 | 74.6 | |
| Taurine |  | µM | -13.3 | -15.4 | 28.9 | -147.0 | 43.7 | |
| Threonine |  | µM | -49.5 | -48.6 | 36.6 | -137.2 | 42.8 | |
| Tryptophan | TRP | µM | -1.24 | -1.44 | 7.80 | -19.6 | 16.8 | |
| Tyrosine | TYR | µM | -12.8 | -13.2 | 17.9 | -62.5 | 38.6 | |
| Valine |  | µM | -41.2 | -41.5 | 72.5 | -183.3 | 173.7 | |
| 1-Methylhistidine | 1-MH | µM | 1.25 | 1.63 | 2.11 | -3.28 | 8.16 | |
| 3-Methylhistidine |  | µM | 0.55 | 0.46 | 5.98 | -17.1 | 20.5 | |
| Relative ADG | rADG_W0-W6_ | kg.d^-1^/kg | 25.0 | 24.0 | 7.0 | 3.5 | 41.8 | |

^1^ Blood variables are differences in concentrations between W0 and W3 calculated as concentrations measured at W3 minus concentration measured at W0. Relative average daily gain (ADG) is calculated as ADG between W0 and W6 divided by body weight at W0. N=136

^2^ CarrU = “Carratelli Units”, where 1 CARRU is equivalent to the oxidizing power of 0.08 mg H_2_O_2_/dL

**Additional Fig. S1** Boxplots for the residuals of the minimal model by hygiene of housing conditions and RFI lines (minimal model = rADG_W6-W0_ ~ FFA + BAP + HAPTO + ILE + TRP+1-MH). The model predicted relative ADG measured during the six weeks of the testing period (rADG_W0-W6_). The variables are changes in the concentrations in plasma between two times W0 and W3 calculated as concentrations at W3 minus concentrations at W0 for free fatty acids or FFA, Biological Antioxidant Potential or BAP, haptoglobin or HAPTO, isoleucine or ILE, tryptophan or TRP, 1-methylhistidine or 1-MH.

**Additional Fig. S2** Boxplots of variables present in at least one selected model. The variables are changes in the concentrations in plasma between two times W0 and W3 calculated as concentrations at W3 minus concentrations at W0 for free fatty acids or FFA, Biological Antioxidant Potential or BAP, haptoglobin or HAPTO, isoleucine or ILE, tryptophan or TRP, 1-methylhistidine or 1-MH, and relative average daily gain calculated between W0 and W6 or rADG_W0-W6._ Outliers (black points) for the boxplot are values outside the range Quartile(Q)3 + 1.5*interquartile-range (IQR), or Q1 – 1.5* IQR. Grey points represent the raw values for each variable, with a random noise added to better distinguish them on the X axis. Therefore, X axis has no particular meaning.

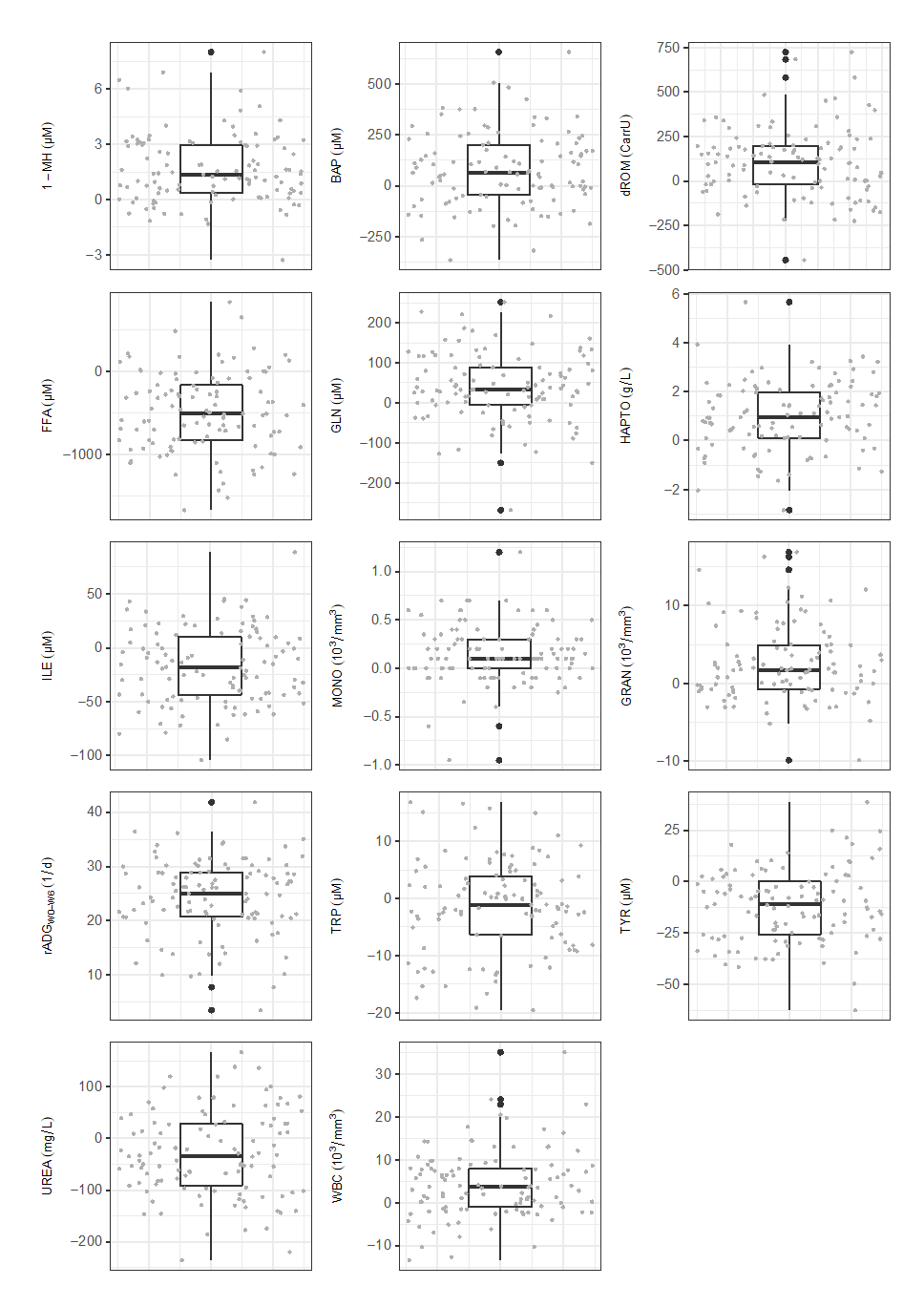

Supplement: Supplementary file 1 — Additional file 1: Table S1. Cross validation of 10 k-folds repeated 10 times for the 11 selected models to predict the relative Average Daily Gain (rADGW0-W6)1. Table S2. Values of the 51 blood variables and relative average daily gain1. Fig. S1. Boxplots for the residuals of the minimal model by hygiene of housing conditions and RFI lines (minimal model = rADGW6-W0 ~ FFA + BAP + HAPTO + ILE + TRP + 1-MH). The model predicted relative ADG measured during the 6 weeks of the testing period (rADGW0-W6). The variables are changes in the concentrations in plasma between two times W0 and W3 calculated as concentrations at W3 minus concentrations at W0 for free fatty acids or FFA, Biological Antioxidant Potential or BAP, haptoglobin or HAPTO, isoleucine or ILE, tryptophan or TRP, 1-methylhistidine or 1-MH. Fig. S2. Boxplots of variables present in at least one selected model. The variables are changes in the concentrations in plasma between two times W0 and W3 calculated as concentrations at W3 minus concentrations at W0 for free fatty acids or FFA, Biological Antioxidant Potential or BAP, haptoglobin or HAPTO, isoleucine or ILE, tryptophan or TRP, 1-methylhistidine or 1-MH, and relative average daily gain calculated between W0 and W6 or rADGW0-W6. Outliers (black points) for the boxplot are values outside the range Quartile(Q)3 + 1.5*interquartile-range (IQR), or Q1–1.5* IQR. Grey points represent the raw values for each variable, with a random noise added to better distinguish them on the X axis. Therefore, X axis has no particular meaning. [file 12917_2021_2872_MOESM1_ESM.docx]
